# TIMELY PERSPECTIVES FROM DIVERSE STAKEHOLDERS ON GENERATIVE AI

**DOI:** 10.1093/geroni/igae098.1921

**Published:** 2024-12-31

**Authors:** Thomas Cudjoe, Nancy Schoenborn, Sato Ashida

**Affiliations:** Johns Hopkins University School of Medicine, Baltimore, Maryland, United States; Johns Hopkins University, Baltimore, Maryland, United States; University of Iowa, Iowa City, Iowa, United States

## Abstract

A core mission of the Johns Hopkins Artificial Intelligence (AI) and Technology Collaboratory for Aging Research is to engage diverse stakeholders. To better understand the perspectives of stakeholders on research priorities and decision-making considerations regarding the development and adoption of AI, we conducted in-depth interviews with stakeholders recruited nationally. We interviewed a total of 49 participants, including older adults/caregivers (n=15) and clinicians (n=15) from urban and rural areas to understand user perspectives. We also interviewed entrepreneurs (n=6), investors (n=5), and payers (n=8)to gain a broader understanding of how generative AI may be integrated into current health service systems. Themes included: 1) Shared and distinct priorities among the stakeholders; most frequently identified priority areas that AI can help address included facilitating social engagement, coordination of care, supporting independence, and treating or preventing common geriatric syndromes like dementia and falls. Older adults and caregivers focused on daily function and caregiving; clinicians focused on monitoring and adherence; health system leaders and payers focused on access, staff burnout, and satisfaction. 2) Participants envisioned multiple roles for AI, including taking over burdensome tasks, making existing tasks more efficient, addressing unmet needs, and performing tasks beyond what is humanly possible. 3) Although perceived benefits outweighed concerns, there were concerns including unintended consequences and suggestions for guardrails. 4) Decisions about AI adoption focused on ease of use, implementation, and cost. Our findings highlight important priorities and considerations from stakeholders for the use of generative AI and opportunities for future research and development.

